# USP30 Beyond Mitophagy: A Context-Dependent Deubiquitinase in Cancer Progression

**DOI:** 10.3390/ijms27209368

**Published:** 2026-10-21

**Authors:** Weijie Liao, Naihan Xu

**Affiliations:** Institute of Key Technologies for Health, School of Food and Drug, Shenzhen Polytechnic University, Shenzhen 518055, China; liao-wj16@tsinghua.org.cn

**Keywords:** USP30, deubiquitinase, mitophagy, mitochondrial quality control, cancer, cancer metabolism, context-dependent regulation, therapy resistance

## Abstract

Ubiquitin-specific protease 30 (USP30) is a mitochondrial outer membrane-associated deubiquitinase best known for antagonizing PTEN-induced kinase 1 (PINK1)/Parkin-dependent ubiquitination and suppressing mitophagy. This established role has linked USP30 to mitochondrial quality control and neurodegenerative diseases. However, emerging studies in cancer have expanded the biological scope of USP30, revealing functions that cannot be explained by mitophagy regulation alone. USP30 regulates diverse cancer-associated substrates through deubiquitination, thereby affecting protein abundance, subcellular localization, protein interactions, and downstream transcriptional programs. These substrate-dependent effects influence mitochondrial dynamics, lipid synthesis, glucose and amino acid metabolism, redox homeostasis, apoptosis, ferroptosis, epithelial–mesenchymal transition, cancer stemness, T-cell exhaustion, and therapy resistance. Although most studies indicate tumor-promoting roles of USP30 in hepatocellular carcinoma, breast cancer, colorectal cancer, oral squamous cell carcinoma, ovarian cancer, bladder cancer, prostate cancer, and leukemia models, context-dependent tumor-restraining activities have also been reported. In this Review, we examine cancer-associated functions of USP30 that extend beyond its established role in PINK1/Parkin-dependent mitophagy, discuss the spatial and substrate-dependent mechanisms underlying these activities, and highlight opportunities and challenges for therapeutic targeting.

## 1. Introduction

Mitochondria are central signaling hubs that coordinate cellular metabolism, oxidative stress responses, apoptosis, innate immunity, and cell fate decisions. During tumorigenesis, mitochondrial functions are extensively rewired to support uncontrolled proliferation, survival under stress, metastatic dissemination, and therapeutic resistance [1]. Mitochondrial homeostasis is therefore not only a housekeeping process but an integral component of cancer cell adaptation and tumor progression.

Among mitochondrial-associated proteins, ubiquitin-specific protease 30 (USP30) has attracted increasing attention. USP30 is anchored to the outer mitochondrial membrane and possesses deubiquitinating activity toward ubiquitinated mitochondrial proteins [2,3]. It was initially identified as a regulator of mitochondrial morphology and later established as a negative regulator of PTEN-induced kinase 1 (PINK1)/Parkin-dependent mitophagy [2,3,4]. Upon mitochondrial damage, PINK1 accumulates on the outer mitochondrial membrane and recruits Parkin, thereby promoting ubiquitination-dependent recognition and clearance of dysfunctional mitochondria. USP30 counteracts this process by removing ubiquitin chains from mitochondrial substrates, limiting mitochondrial ubiquitination and mitophagic flux [3,5,6,7,8]. By regulating mitochondrial ubiquitin signaling, USP30 plays an important role in mitochondrial quality control and has been implicated in neurodegenerative diseases, particularly Parkinson’s disease and related disorders characterized by mitochondrial dysfunction [9,10]. Indeed, early pharmacological interest in USP30 inhibition was largely driven by the possibility of enhancing mitophagy in these settings [11,12,13,14,15].

Cancer-focused studies have substantially broadened the known biological functions of USP30. In tumor cells, USP30 has been implicated not only in mitochondrial quality control but also mitochondrial dynamics, metabolic reprogramming, apoptosis, ferroptosis, epithelial–mesenchymal transition (EMT), cancer stemness, immune-cell dysfunction, and therapeutic resistance. Several USP30 substrates implicated in cancer biology are not established components of the PINK1/Parkin mitophagy pathway but instead participate in distinct functional programs relevant to tumor adaptation. In metabolic reprogramming, USP30 has been reported to regulate ATP citrate lyase (ACLY) and fatty acid synthase (FASN) in lipid synthesis, hexokinase 2 (HK2) in glucose metabolism, and fat mass and obesity-associated protein (FTO) in serine biosynthesis [16,17,18]. Mitochondrial dynamics and homeostasis have been linked to substrates such as dynamin-related protein 1 (DRP1) and translocase of outer mitochondrial membrane 40 (TOMM40) [19,20], whereas catalase and glutathione peroxidase 4 (GPX4) connect USP30 to redox control and regulate cell death [21,22]. Other reported substrates regulate malignant growth and plasticity through distinct mechanisms, including c-Myc in tumor-cell growth and metabolic demand, and Snail and S100 calcium-binding protein A6 (S100A6) in invasion and dissemination [23,24,25]. In contrast, under specific stress conditions, nuclear USP30-dependent regulation of T-cell factor/lymphoid enhancer-binding factor 1 (TCF/LEF1) has been reported to restrain Wnt-driven stemness and therapeutic resistance [26]. These findings indicate that the biological effects of USP30 cannot be understood solely through its regulation of mitophagy. Instead, they depend on the specific substrates, subcellular compartments, and cellular programs engaged in each cancer context. Importantly, the functional consequences of USP30 are not determined by tumor lineage alone. For example, in breast cancer, USP30 has been reported to promote malignant progression through Snail and TOMM40 in some settings, whereas nutrient stress-induced nuclear USP30 suppresses TCF/LEF1-dependent stemness and metastatic phenotypes in others [20,24,26]. Together with the tumor-restraining phenotype reported in glioblastoma [27], these observations suggest that subcellular localization, substrate availability, cellular state, and stress conditions are critical determinants of USP30 function.

Previous reviews have provided valuable overviews of USP30 structure, its roles in organelle quality-control, its broader disease associations, and the development of pharmacological inhibitors [28,29]. However, these frameworks have largely been organized around the established role of USP30 in mitochondrial and peroxisomal quality control and do not fully capture the mechanistic diversity emerging from recent cancer studies. In particular, accumulating evidence now implicates USP30 in the regulation of substrates involved in metabolism, redox control, cell death, malignant plasticity, therapy resistance, and immune-cell dysfunction. Several of these functions are spatially and mechanistically distinct from PINK1/Parkin-dependent mitophagy, and some produce opposing biological outcomes depending on tumor lineage, subcellular localization, substrate availability, and cellular stress. These findings raise questions that are not readily addressed by a mitophagy-centered framework, including why USP30 is tumor-promoting in most cancer models yet tumor-restraining in selected contexts, how distinct substrate modules determine cancer-specific USP30 dependency, and whether pharmacological USP30 inhibition can be translated safely across heterogeneous tumor and immune-cell states.

In this review, we do not simply catalogue recently reported USP30-associated cancer pathways. Instead, we distinguish the established role of USP30 in antagonizing PINK1/Parkin-dependent mitochondrial ubiquitin signaling from its peroxisomal and cancer-associated activities. Peroxisomal pexophagy is considered a distinct organelle quality-control function, whereas nuclear and other cancer-related effects are examined in relation to their subcellular localization, substrate repertoire, and functional consequences. On this basis, we organize the emerging cancer biology of USP30 around four interrelated dimensions—subcellular localization, substrate identity, functional output, and tumor context. We then examine how these dimensions shape metabolic plasticity, mitochondrial adaptation, regulated cell death, malignant progression, therapeutic resistance, and tumor immunity. Finally, we discuss how this framework may help define biomarker-guided USP30 dependency and the conditions under which USP30-regulated pathways could represent therapeutic vulnerabilities.

## 2. Established Functions of USP30 in Organelle Quality Control

### 2.1. Molecular Features, Subcellular Localization, and Regulation of USP30

USP30 is a member of the ubiquitin-specific protease family and is best characterized as a mitochondria-associated deubiquitinating enzyme. It is predominantly anchored to the outer mitochondrial membrane by its N-terminal transmembrane region, with the catalytic domain exposed to the cytosol [2,30,31]. This topology enables USP30 to access ubiquitinated cytosol-facing domains of outer mitochondrial membrane proteins and directly modulate mitochondrial ubiquitin signaling [2,3].

Although USP30 is classically regarded as a mitochondrial deubiquitinase, it is not restricted to mitochondria. A fraction of endogenous USP30 is also associated with peroxisomes, indicating that its membrane-associated functions extend to multiple organelle quality-control systems [32,33]. In addition, a context-dependent nuclear pool of USP30 has been reported in breast cancer cells under metabolic stress. This observation raises the possibility that USP30 may regulate nuclear substrates in a manner that is spatially and mechanistically distinct from its mitochondrial activities [26]. These spatially distinct pools provide a potential basis for both mitochondria-dependent and mitochondria-independent functions.

Biochemical and structural studies have revealed distinctive catalytic and ubiquitin-chain recognition properties of USP30. Its catalytic activity depends on a unique catalytic triad composed of Cys77, His452, and Ser477, which contributes to its selective recognition and processing of specific ubiquitin linkages [30,31]. USP30 preferentially cleaves Lys6-linked ubiquitin chains, whereas its activity toward other ubiquitin linkages appears more limited and may depend on the substrate or cellular context [5,17,26,30,31]. Structural analyses of USP30 bound to monoubiquitin or Lys6-linked diubiquitin further demonstrate that USP30 contains specialized ubiquitin-binding interfaces that orient Lys6-linked chains for efficient cleavage [30,31]. This Lys6 linkage preference provides an important biochemical basis for USP30 activity toward ubiquitinated mitochondrial substrates.

The abundance, subcellular localization, and activity of USP30 are further regulated by post-translational modifications. Parkin monoubiquitinates USP30 at Lys235, Lys289, and Lys310. Monoubiquitinated USP30 retains catalytic activity and appears relatively stable; however, the functional consequences of these modifications remain incompletely understood [3,30]. Inhibitor of nuclear factor kappa-B kinase subunit beta (IKKβ) directly phosphorylates USP30 at Ser210 and Ser364, thereby increasing USP30 stability [16]. More recently, Ser104 was identified as a context-dependent phosphorylation site regulated by mechanistic target of rapamycin complex 1 (mTORC1) signaling. Under nutrient stress, reduced Ser104 phosphorylation promotes USP30 translocation to the nucleus, linking nutrient sensing to the subcellular redistribution of USP30 [26]. USP30 activity is also influenced by PINK1-mediated phosphorylation of ubiquitin at Ser65. Ser65-phosphorylated ubiquitin, particularly within Lys6-linked chains, is less efficiently hydrolyzed by USP30 [30,31,34,35].

In addition to post-translational mechanisms, USP30 expression can be regulated by the antisense long noncoding RNA USP30-AS1 in a cancer-type-dependent manner. In acute myeloid leukemia (AML), USP30-AS1 is highly expressed and is associated with poor prognosis. Functional studies have shown that USP30-AS1 promotes AML-cell viability and suppresses apoptosis, at least in part through cis-regulation of USP30. This regulatory relationship was associated with changes in H3K4me3 and H3K27ac at the USP30 locus [36]. Similarly, in cervical cancer, USP30-AS1 is likewise upregulated and enhances USP30 expression through a miR-2467-3p/FUS-dependent mechanism. Increased USP30 subsequently stabilizes β-catenin and activates Wnt/β-catenin signaling, thereby promoting tumor-cell proliferation, migration, and invasion [37]. These findings identify USP30-AS1 as an additional upstream regulator of USP30 and suggest that noncoding RNA-dependent control of USP30 abundance may contribute to heterogeneity in USP30 expression and function across cancer types.

**Figure 1:**
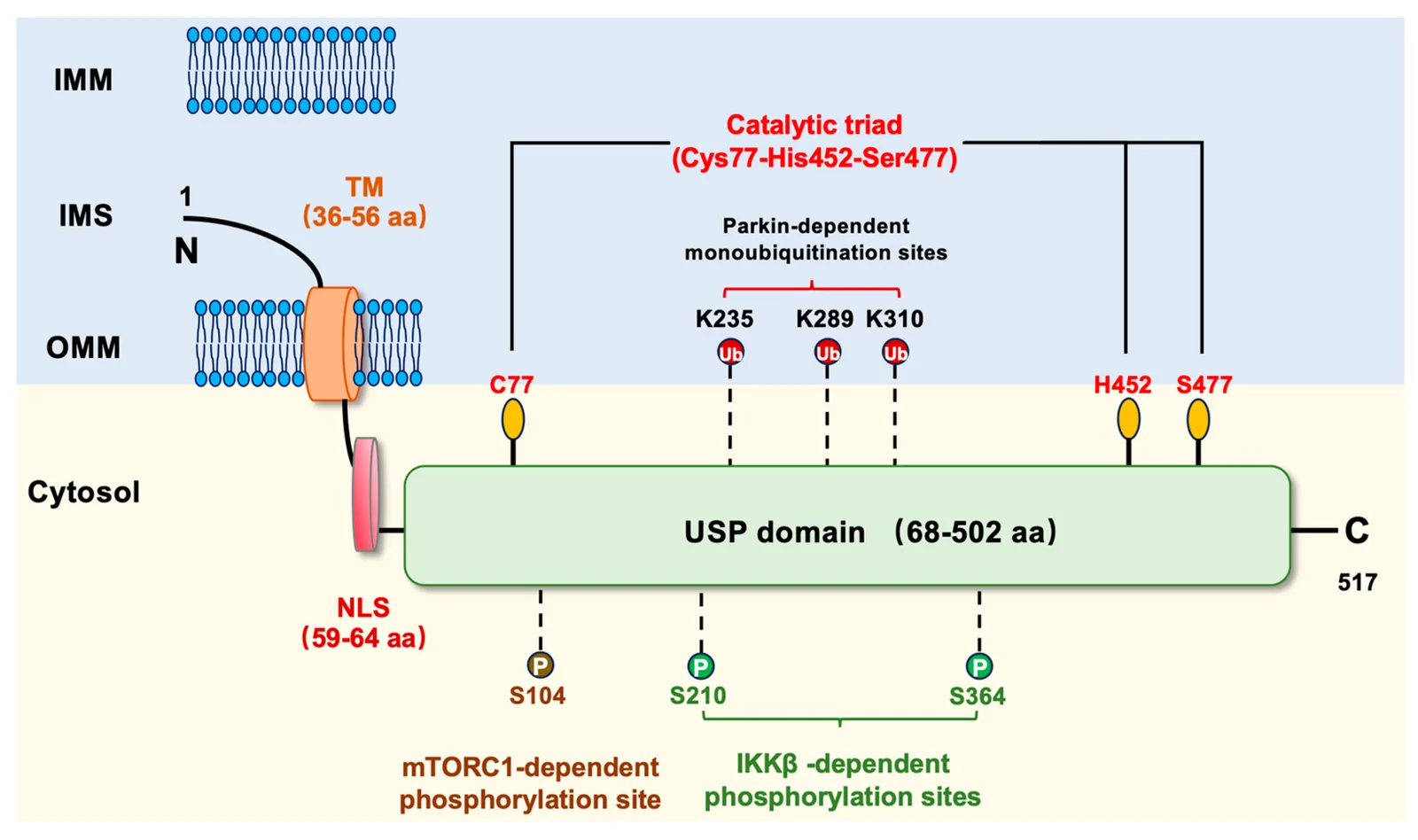
Molecular architecture, membrane topology, and regulatory sites of USP30. The schematic shows the N-terminal transmembrane segment, nuclear localization signal, USP catalytic domain and catalytic triad, together with reported Parkin-dependent monoubiquitination and mTORC1- or IKKβ-dependent phosphorylation sites. Solid connector lines group the catalytic-triad residues, whereas dashed connector lines indicate reported post-translational modification sites. OMM, outer mitochondrial membrane; IMS, intermembrane space; TM, transmembrane segment; NLS, nuclear localization signal; USP30, ubiquitin-specific protease 30; mTORC1, mechanistic target of rapamycin complex 1; IKKβ, inhibitor of nuclear factor kappa-B kinase subunit beta.

Together, these structural, spatial, and regulatory features provide a molecular basis for the diverse functions of USP30 (Figure 1). The N-terminal membrane anchor, cytosolic catalytic domain, linkage-selective ubiquitin recognition, post-translational regulation, and non-coding RNA-mediated control of expression collectively determine whether USP30 acts, which substrates it engages, and how its activity is translated into organelle-specific or cancer-associated phenotypes.

### 2.2. USP30 in Mitochondrial Quality Control

USP30 is best known for its established role in mitochondrial quality control, particularly as an antagonist of PINK1/Parkin-dependent mitophagy. Upon mitochondrial damage, USP30 counteracts the accumulation of ubiquitin signals generated on the outer mitochondrial membrane and thereby limits mitophagic signaling [3,5,6]. In addition to mitophagy, USP30 regulates mitochondrial morphology and dynamics through effects on proteins involved in mitochondrial fission and fusion [2,19,38,39].

Under basal conditions, PINK1 is imported into healthy mitochondria and rapidly degraded. Upon mitochondrial depolarization, PINK1 accumulates on the outer mitochondrial membrane and phosphorylates ubiquitin at Ser65 and Parkin within its ubiquitin-like domain at Ser65, thereby activating Parkin and promoting widespread ubiquitination of outer mitochondrial membrane proteins [34,40,41,42]. These ubiquitin signals recruit autophagy receptors and initiate lysosomal clearance of damaged mitochondria. USP30 counteracts this process by removing Parkin-deposited ubiquitin chains from mitochondrial substrates, thereby limiting the amplification of mitophagy-promoting ubiquitin signals [3,5,6]. USP30 overexpression suppresses mitochondrial ubiquitination and Parkin-dependent mitophagy, whereas USP30 knockdown, knockout, or pharmacological inhibition enhances mitochondrial ubiquitination and accelerates mitophagic flux [3,11,12,13]. USP30 is therefore thought to function as a threshold-setting enzyme for mitophagy initiation. By deubiquitinating outer mitochondrial membrane substrates, USP30 restricts ubiquitin accumulation near sites of PINK1 stabilization and dampens Parkin-dependent signal amplification [3,6,10].

The mitophagy-suppressive function of USP30 has been widely studied in diseases involving mitochondrial dysfunction, especially Parkinson’s disease and neurological injury. Genetic depletion or pharmacological inhibition of USP30 can restore mitophagy in models carrying pathogenic Parkin mutations, improve mitochondrial integrity, reduce oxidative stress, and protect dopaminergic neurons from mitochondrial stress [3,14,15]. In animal models, USP30 loss or inhibition attenuates dopaminergic neuronal loss and improves motor phenotypes [14]. Similar protective effects have been reported in traumatic brain injury and subarachnoid hemorrhage models, where USP30 inhibition promotes neuronal mitophagy and reduces mitochondrial damage, apoptosis, oxidative stress, and neuroinflammation [43,44].

USP30 also contributes to mitochondrial quality control by regulating mitochondrial dynamics. Fusion maintains mitochondrial network integrity and content exchange, whereas fission segregates damaged fragments for mitophagic removal [39,45,46,47]. Early studies showed that USP30 depletion induced elongated, interconnected mitochondrial networks in a mitofusin-dependent manner, and this phenotype was rescued by catalytically active USP30 [2,38]. USP30 inhibition or depletion also increases non-degradative ubiquitination of mitofusin 1 (MFN1) and mitofusin 2 (MFN2), enhancing mitofusin-dependent fusion [38]. These findings indicate that USP30 controls mitochondrial morphology by editing both degradation-associated and non-proteolytic ubiquitin signals on the fusion machinery [5,38].

The effects of USP30 on mitochondrial dynamics are substrate- and context-dependent. In hepatocellular carcinoma, USP30 promotes fission by stabilizing DRP1 through counteracting glyceronephosphate O-acyltransferase (GNPAT)-dependent K48-linked ubiquitination, thereby maintaining a fragmented mitochondrial state [19]. USP30 inhibition reduces DRP1 levels, shifts mitochondria toward fusion, and suppresses hepatocellular carcinoma cell proliferation and tumor progression [19]. Conversely, under oxygen–glucose deprivation/reperfusion stress, USP30 may protect MFN2 from ubiquitination and degradation, preserving fusion and limiting excessive fragmentation [48]. USP30 may also affect mitochondrial dynamics indirectly through the translocase of the outer mitochondrial membrane (TOM) complex and translocon components, which are USP30-sensitive substrates and may influence PINK1 import, stabilization, and mitophagy initiation [10,49,50,51]. Thus, USP30 links mitochondrial morphology, import-dependent damage sensing, and mitophagy control through context-specific deubiquitination.

### 2.3. USP30 in Peroxisomal Quality Control

Peroxisomes are metabolically active organelles involved in very-long-chain fatty acid oxidation, ether-lipid synthesis, hydrogen peroxide metabolism, and cellular redox homeostasis. Their abundance and functional integrity are maintained partly through pexophagy, the selective autophagic removal of damaged, dysfunctional, or superfluous peroxisomes [52,53]. In addition to its mitochondrial localization, a fraction of endogenous USP30 is targeted to peroxisomal membranes, where it acts as a negative regulator of basal pexophagy [32,33].

At peroxisomes, USP30 counteracts ubiquitin-dependent peroxisomal turnover by opposing the activity of the peroxisomal E3 ubiquitin ligase peroxisomal biogenesis factor 2 (PEX2) [54,55]. By limiting the accumulation of ubiquitin signals on peroxisomal proteins, including ATP-binding cassette subfamily D member 3/peroxisomal membrane protein 70 (ABCD3/PMP70) and proteins associated with the peroxisomal import machinery, USP30 reduces autophagy-receptor recruitment and helps preserve basal peroxisome abundance. Consistently, USP30 overexpression suppresses starvation-induced peroxisome degradation, whereas genetic depletion of USP30 promotes basal and stress-induced pexophagy. Pharmacological inhibition of USP30 similarly enhances peroxisomal turnover, indicating that its catalytic activity is required to restrain pexophagy [12,32,33].

Notably, USP30-dependent regulation of pexophagy appears to occur independently of the PINK1/Parkin pathway. This finding indicates that USP30 can suppress selective autophagy through distinct organelle-specific ubiquitination systems, rather than acting solely within mitochondrial quality control. Although the relevance of USP30-dependent pexophagy to cancer remains insufficiently defined, this pathway may be biologically important given the dependence of tumor cells on organelle metabolism, lipid remodeling, redox balance, and metabolic plasticity.

**Figure 2:**
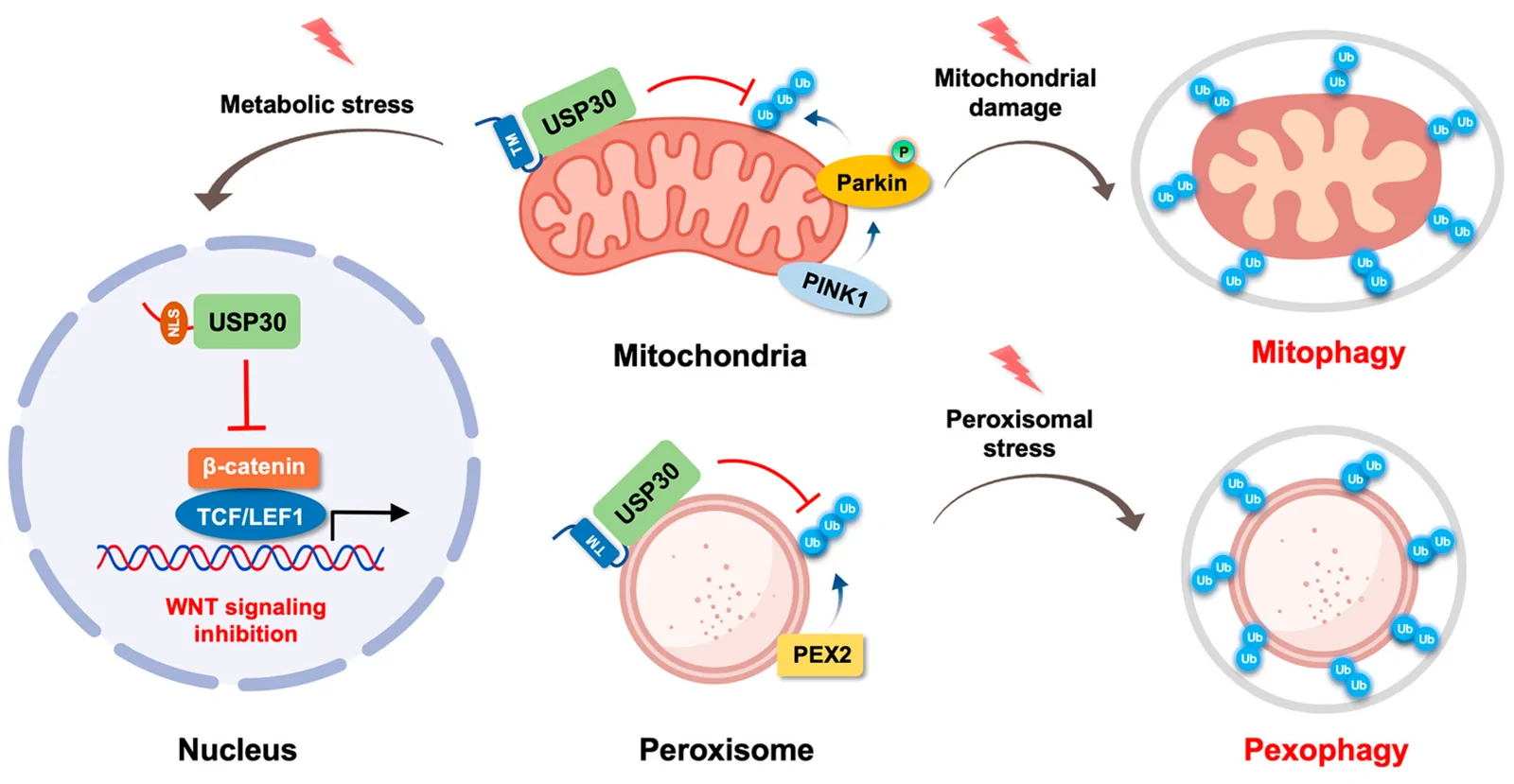
Subcellular distribution of USP30 and its distinct functions. The majority of USP30 localizes to the outer mitochondrial membrane, where it antagonizes PINK1/Parkin-mediated mitophagy. A fraction of USP30 is also targeted to peroxisomes, where it counteracts ubiquitin-dependent peroxisomal turnover by opposing the activity of the peroxisomal E3 ubiquitin ligase PEX2. Under metabolic stress conditions, such as nutrient deprivation, a small pool of USP30 can translocate to the nucleus via its N-terminal nuclear localization signal (NLS), where it directly deubiquitinates TCF/LEF1 and suppresses Wnt signaling. Arrows indicate the direction of regulatory or trafficking processes, whereas blunt-ended lines indicate inhibitory effects. PINK1, PTEN-induced kinase 1; PEX2, peroxisomal biogenesis factor 2; TCF/LEF1, T-cell factor/lymphoid enhancer-binding factor 1.

Together, studies of mitochondrial and peroxisomal USP30 establish a spatially organized model of organelle quality control. The mitochondrial pool regulates PINK1/Parkin-dependent mitophagy and mitochondrial dynamics, whereas the peroxisomal pool restrains PINK1/Parkin-independent pexophagy. A context-dependent nuclear pool, in contrast, may regulate nuclear substrates or transcriptional programs independently of these membrane-associated quality-control pathways. These spatially distinct activities provide an important mechanistic foundation for understanding the broader functions of USP30 in cancer (Figure 2).

## 3. Cancer-Associated Functions of USP30 Beyond Mitophagy

Although USP30 is best established as a regulator of mitochondrial ubiquitin signaling and mitophagy, its cancer-associated functions extend well beyond this pathway. In tumor and immune cells, USP30 engages spatially and functionally diverse substrates involved in metabolism, mitochondrial dynamics, redox control, apoptosis, ferroptosis, epithelial–mesenchymal transition (EMT), stemness, therapy resistance, and immune regulation (Table 1). As summarized in Table 1, these functions are distributed across multiple subcellular compartments and biological processes. They are not mechanistically uniform: some arise from direct deubiquitination and stabilization of substrates, whereas others involve changes in substrate localization, activity, protein interactions, or transcriptional output. Consequently, the biological effects of USP30 can be tumor-promoting or tumor-restraining depending on substrate identity, subcellular localization, tumor lineage, and cellular state.

### 3.1. USP30 and Cancer Metabolic Reprogramming

Metabolic reprogramming is a defining feature of cancer. Tumor cells alter glucose metabolism, lipid synthesis, amino acid utilization, and redox pathways to sustain proliferation and survive nutrient stress [60,61,62]. Increasing evidence suggests that USP30 contributes to this metabolic plasticity by deubiquitinating multiple metabolic regulators [16,17,18]. Rather than acting through a single metabolic pathway, USP30 regulates metabolic proteins through substrate-dependent mechanisms that can influence protein abundance, subcellular localization, molecular interactions, and enzymatic activity.

One of the clearest links between USP30 and cancer metabolism has been reported in hepatocellular carcinoma [16]. In human hepatocellular carcinoma (HCC), the IKKβ-USP30-ACLY axis is activated. IKKβ phosphorylates and stabilizes USP30, thereby enhancing USP30-mediated deubiquitination and stabilization of ATP citrate lyase (ACLY) and fatty acid synthase (FASN). ACLY connects mitochondrial citrate metabolism with cytosolic lipid synthesis, whereas FASN catalyzes de novo fatty acid synthesis [63,64,65,66]. Together, ACLY and FASN support membrane biogenesis, lipid signaling, and rapid tumor-cell proliferation. Through this mechanism, USP30 acts as a bridge connecting inflammatory kinase signaling, lipid metabolism, and liver cancer progression.

USP30 has also been implicated in glucose metabolism. Hexokinase 2 catalyzes the first step of glycolysis and is frequently upregulated in many cancer types [67,68,69]. In addition to its catalytic function, HK2 can associate with voltage-dependent anion channel 1 (VDAC1) on the outer mitochondrial membrane, enhancing glycolytic efficiency and protecting cells from apoptosis [17,69,70]. A recent study showed that USP30 deubiquitinates HK2 and regulates its stability, and identified lysine 144 as a critical residue in this regulatory process. Mutation of this site increases HK2 stability, enhances its mitochondrial localization and interaction with VDAC1, and augments hexokinase activity [17]. Through this mechanism, USP30 promotes glycolytic flux, mitochondrial association of HK2, and tumor progression.

USP30 also regulates amino acid metabolic programs. In colorectal cancer, USP30 senses serine/glycine availability and regulates serine biosynthesis by deubiquitinating and stabilizing FTO, an RNA demethylase [18,71,72,73]. FTO modulates the phosphoglycerate dehydrogenase (PHGDH)/phosphoserine aminotransferase 1 (PSAT1) axis, thereby promoting serine biosynthesis and supporting metabolic adaptation during nutrient stress [18]. Since serine and glycine metabolism provides one-carbon units, nucleotide precursors, and reducing equivalents, the USP30–FTO–PHGDH/PSAT1 axis expands the role of USP30 from a mitochondrial deubiquitinase to a nutrient-responsive regulator of anabolic metabolism [74]. Notably, sodium 2,2-dichloroacetate (DCA), a potential USP30 inhibitor, suppresses serine synthesis and colorectal tumor growth, suggesting that USP30 may be therapeutically targetable in tumors dependent on serine/glycine metabolic pathways.

**Figure 3:**
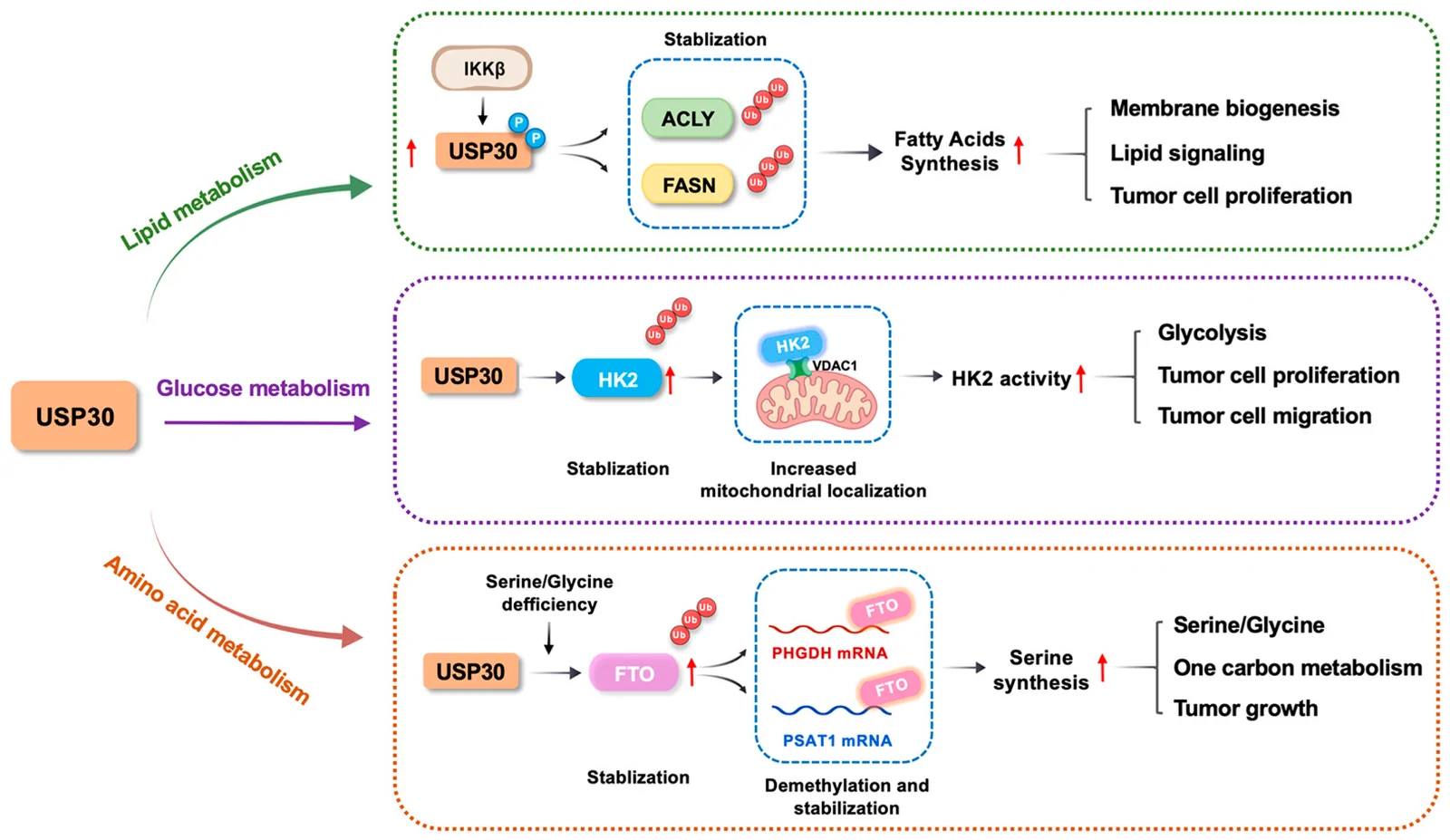
USP30-associated metabolic programs in cancer. The schematic summarizes reported links between USP30 and three metabolic modules: lipid metabolism through ACLY/FASN, glucose metabolism through HK2, and serine/glycine metabolism through the FTO–PHGDH/PSAT1 axis. These pathways are derived from distinct experimental and tumor contexts and are not intended to imply simultaneous regulation within a single tumor. Arrows indicate the direction of regulatory effects, and upward arrows denote increased protein abundance, activity, or pathway output. ACLY, ATP citrate lyase; FASN, fatty acid synthase; HK2, hexokinase 2; FTO, fat mass and obesity-associated protein; PHGDH, phosphoglycerate dehydrogenase; PSAT1, phosphoserine aminotransferase 1.

Together, these studies indicate that USP30 influences cancer metabolic plasticity through substrate-specific deubiquitination of regulators involved in lipid, glucose, and amino acid metabolism (Figure 3). The molecular consequences are not limited to changes in protein abundance. In the case of HK2, altered ubiquitination is also associated with mitochondrial localization, VDAC1 interaction, and enzymatic activity [17]. However, whether ACLY, HK2 and FTO are functionally integrated within the same tumor type remains unclear. It is also unknown whether USP30 defines a coordinated metabolic dependency or instead regulates distinct metabolic modules in a context-specific manner.

From a translational perspective, these observations raise the testable hypothesis that USP30 inhibition could perturb more than one metabolic program in tumors in which multiple USP30-regulated substrates are functionally relevant. This possibility has not yet been demonstrated within a single tumor model and requires direct experimental validation. Whether such multi-pathway metabolic disruption could be therapeutically exploited in combination with chemotherapy, targeted therapy, or dietary metabolic interventions requires further validation.

### 3.2. USP30 in EMT, Metastasis, and Malignant Plasticity

Epithelial–mesenchymal transition (EMT) and malignant plasticity enable cancer cells to acquire migratory, invasive, and survival capacities [75,76,77]. Emerging evidence suggests that USP30 contributes to malignant plasticity through deubiquitination-dependent regulation of proteins involved in phenotype switching, redox adaptation, and metastatic dissemination.

A well-characterized example is the USP30-Snail axis in breast cancer. Snail is a central EMT transcription factor that represses E-cadherin and promotes metastatic behavior [78,79,80]. USP30 directly interacts with Snail and removes K48-linked polyubiquitin chains, thereby reducing its proteasomal degradation [24]. Stabilized Snail enhances EMT, migration, invasion, proliferation, and tumor growth. In addition, USP30 reduces breast cancer cell sensitivity to paclitaxel, suggesting that the USP30–Snail axis contributes not only to metastasis but also to chemotherapy resistance.

USP30 has also been implicated in ovarian cancer metastasis through S100A6 stabilization. In this setting, a forkhead box F1 (FOXF1)-high-mobility group AT-hook 2 (HMGA2)-USP30-S100A6 regulatory axis has been identified [25]. FOXF1 promotes HMGA2-mediated recruitment of USP30 to S100A6 (S100 calcium-binding protein A6), allowing USP30 to deubiquitinate and stabilize S100A6. As a calcium-binding protein involved in cytoskeletal remodeling, motility, invasion, and metastasis [25,81,82,83], S100A6 supports ovarian cancer dissemination when protected from degradation by USP30. This study highlights that USP30 substrate specificity may be shaped by adaptor proteins or tumor-specific interaction networks.

In bladder cancer, USP30 regulates malignant behavior through a redox-related mechanism involving catalase. Parkin interacts with and ubiquitinates catalase, promoting its proteasomal degradation and increasing intracellular reactive oxygen species (ROS) levels [21,84,85]. Conversely, USP30 directly interacts with catalase and removes its ubiquitin modifications, thereby increasing catalase stability. The catalytically inactive USP30-C77S mutant fails to stabilize catalase, supporting a direct requirement for USP30 deubiquitinase activity. Through stabilization of catalase, USP30 reduces intracellular ROS levels and promotes bladder cancer cell proliferation and migration. These findings identify catalase as a deubiquitination substrate of USP30 and establish a Parkin–USP30–catalase axis in bladder cancer [21].

Together, these studies suggest that USP30 promotes malignant plasticity by stabilizing proteins that control EMT, ROS buffering, cytoskeletal remodeling, and metastatic dissemination. However, how USP30 accesses non-mitochondrial substrates such as Snail and S100A6 remains unclear. Future spatial proteomic and ubiquitinomic studies are needed to define the subcellular USP30 pools, adaptor proteins, substrate-recognition mechanisms, and ubiquitin-chain specificities that determine USP30-dependent metastatic programs.

### 3.3. USP30 in Cancer Cell Survival and Regulated Cell Death

Cancer cells must maintain mitochondrial fitness while avoiding excessive oxidative stress and cell death. Beyond its critical role in mitochondrial quality control, USP30 can influence apoptotic threshold, redox homeostasis, and regulated cell death through cancer-associated substrates and stress-response pathways [6,22,56].

USP30 has been linked to intrinsic apoptosis through BCL-2-associated X protein (BAX)/BCL-2 antagonist/killer 1 (BAK)-dependent mechanisms. Early studies showed that USP30 deubiquitinates mitochondrial Parkin substrates and restricts apoptotic cell death, while USP30 depletion sensitizes cancer cells to BCL-2 homology 3 (BH3) mimetics [6]. These findings suggest that USP30 helps maintain a higher mitochondrial apoptotic threshold. Consistently, Aumdubin, an auranofin derivative identified as a USP30 inhibitor, induces BAX-dependent mitochondrial apoptosis in lung cancer cells both in vitro and in vivo [56]. These data indicate that pharmacological inhibition of USP30 can promote mitochondrial apoptosis and may cooperate with therapies that activate the intrinsic apoptotic pathway. USP30 has also been linked to pro-survival protein kinase B (AKT)/mechanistic target of rapamycin (mTOR) signaling. In a leukemia-related model, USP30 overexpression antagonized Parkin activity, sustained AKT/mTOR signaling, and reduced apoptosis [57,86]. These findings suggest that USP30 may support tumor-cell survival through both mitochondrial ubiquitin regulation and downstream growth-survival signaling, providing a potential rationale for combining USP30 inhibition with AKT/mTOR pathway inhibitors.

Ferroptosis is an iron-dependent form of regulated cell death driven by lipid peroxidation [87,88,89]. GPX4 is a central suppressor of ferroptosis because it detoxifies lipid hydroperoxides and protects cells from oxidative membrane damage [88,90,91]. A selenium-containing compound, 8 m, was reported to induce ferroptosis in advanced prostate cancer cells by targeting the USP30-GPX4 axis. Mechanistically, USP30 deubiquitinates and stabilizes GPX4, whereas inhibition of USP30 by 8 m increases GPX4 ubiquitination and promotes its degradation. The resulting loss of GPX4 enhances lipid peroxidation and ferroptotic cell death. These findings identify GPX4 as a deubiquitination substrate of USP30 in prostate cancer cells and suggest that pharmacological disruption of the USP30–GPX4 axis may provide a strategy for inducing ferroptosis [22]. If validated more broadly, USP30 inhibition could represent a strategy to sensitize tumors to ferroptosis inducers, lipid peroxidation, or radiotherapy.

Together, these studies indicate that USP30 influences tumor-cell survival and death through regulation of mitochondrial apoptosis, AKT/mTOR-associated survival signaling, and ferroptosis-related pathways. Thus, USP30 functions as a context-dependent modulator of cancer cell death, with therapeutic potential that may extend across multiple regulated cell-death programs.

### 3.4. USP30 in Cancer Stemness and Therapeutic Resistance

Cancer stemness contributes to tumor initiation, metastasis, recurrence, immune evasion, and resistance to therapy [62,92,93,94]. Several USP30-regulated pathways may reinforce therapy resistance. The USP30–Snail axis described above also contributes to paclitaxel resistance in breast cancer [24]. USP30 also promotes tamoxifen resistance through deubiquitination and stabilization of TOMM40, a component of the mitochondrial outer membrane translocase complex [20]. Stabilized TOMM40 may enhance mitochondrial protein import, mitochondrial homeostasis, and survival under endocrine therapy pressure, connecting mitochondrial protein import machinery to endocrine resistance [20,51]. In acute myeloid leukemia (AML), forkhead box M1 (FOXM1) was identified as a USP30 substrate. USP30 deubiquitinates and stabilizes FOXM1, thereby promoting AML-cell proliferation and survival. The USP30–FOXM1 axis also enhanced cytarabine resistance, whereas USP30 depletion suppressed the growth of cytarabine-resistant AML cells [58].

Importantly, the functional effects of USP30 are not uniformly tumor-promoting. Breast cancer illustrates this context dependence particularly clearly. USP30 can promote malignant progression through stabilization of Snail and TOMM40, contributing to EMT, metastasis, and resistance to paclitaxel or tamoxifen [20,24]. In contrast, under nutrient stress, reduced mTORC1-dependent phosphorylation promotes nuclear accumulation of USP30. Nuclear USP30 directly deubiquitinates TCF/LEF1 at K379 and K382, disrupting CREB-binding protein (CBP)/P300 recruitment to the β-catenin/LEF1 complex and suppressing Wnt-driven stemness, adriamycin resistance, and metastasis [26]. Notably, this mechanism differs from the stabilization of several other USP30 substrates, because TCF/LEF1 deubiquitination primarily alters transcriptional-complex assembly and transcriptional output rather than substrate abundance. The coexistence of tumor-promoting and tumor-restraining functions within the same cancer lineage indicates that the biological outcome of USP30 is determined not simply by tumor type, but by its subcellular localization, available substrates, and cellular state. A tumor-restraining phenotype has also been reported in glioblastoma, where USP30 expression was reduced in tumor samples and ectopic USP30 expression suppressed proliferation, DNA synthesis, and colony formation in U87 cells [27]. However, unlike the mechanistically defined nuclear USP30–TCF/LEF1 pathway, the relevant USP30 substrate and downstream mechanism in glioblastoma remain unknown.

Therapeutically, USP30 inhibition may be most effective in combination with other treatments, such as endocrine therapy, chemotherapy, BH3 mimetics, metabolic inhibitors, ferroptosis inducers, radiotherapy, or targeted therapy [6,17,18,20,22,24,56]. However, the potential tumor-suppressive function of nuclear USP30 in Wnt/β-catenin signaling cautions against viewing USP30 as a universal oncogenic dependency. Biomarker-based stratification will be important to identify tumors that are dependent on USP30-regulated substrates or pathways, while avoiding contexts in which USP30 restrains stemness or tumor progression.

### 3.5. USP30 in Cancer Immunity and the Tumor Microenvironment

The role of USP30 in cancer immunity and the tumor microenvironment remains less well-characterized. Nevertheless, mitochondria are central regulators of innate immune signaling, ROS production, inflammatory responses, and immunogenic cell death [95,96,97,98]. Given that USP30 regulates mitophagy and mitochondrial stress responses, it may influence interactions between tumor cells and immune cells within the tumor microenvironment [59,95].

Recent evidence suggests that USP30 regulates antitumor T-cell function. In exhausted tumor-infiltrating CD8+ T cells, prolonged antigen stimulation upregulates USP30 through T-cell receptor (TCR)/nuclear factor of activated T cells 1 (NFAT1) signaling [59]. Elevated USP30 impairs mitochondrial quality control, reduces T-cell effector function, and promotes exhaustion. Conversely, USP30 inhibition restores mitophagy, improves CD8+ T-cell activity, enhances antitumor immunity, and suppresses tumor growth. Although this mechanism remains related to mitophagy, it has important cancer-immunological implications because USP30 inhibition may reinvigorate exhausted T cells within the tumor microenvironment.

USP30 may also affect tumor immunity indirectly through mitochondrial damage signaling and metabolism. Damaged mitochondria can release mitochondrial DNA, cardiolipin, or other danger-associated molecular patterns that activate innate immune pathways, including cyclic GMP–AMP synthase–stimulator of interferon genes (cGAS-STING) and inflammatory cytokine signaling [95,96,99]. By modulating mitochondrial quality control, USP30 may alter the persistence of damaged mitochondria and thereby influence inflammatory signaling in tumor or immune cells. In parallel, USP30-regulated metabolic programs, including glycolysis, lipid synthesis, glutamine, and serine/glycine metabolism, may shape nutrient competition, redox balance, and immunomodulatory metabolite production within the tumor microenvironment [63,74,100].

These findings raise the possibility that USP30 inhibitors could exert dual antitumor effects by directly impairing tumor-cell adaptation while enhancing antitumor immune function. In particular, USP30 inhibition may have potential in combination with immune checkpoint blockade, such as programmed cell death protein 1/programmed death-ligand 1 (PD-1/PD-L1) or cytotoxic T-lymphocyte-associated protein 4 (CTLA-4) inhibitors. However, this possibility requires careful validation, as USP30 inhibition may produce distinct or even opposing effects in tumor cells, exhausted T cells, myeloid cells, stromal cells, and normal tissues. Future studies should use immunocompetent tumor models, single-cell profiling, and spatial analyses to define the cell type-specific consequences of USP30 inhibition within the tumor microenvironment.

### 3.6. Pharmacological Targeting of USP30

The enzymatic activity and defined catalytic architecture of USP30 have facilitated the development of chemically diverse inhibitors and molecular probes. Early selective USP30 inhibitors were primarily developed to enhance mitophagy in models of mitochondrial dysfunction [11,12,13,14]. More recently, structure-guided studies have yielded benzosulfonamide inhibitors, cyanopyrrolidine-containing covalent compounds, non-covalent small molecules, and activity-based probes, expanding the available toolkit for biochemical validation and therapeutic development [101,102,103,104].

Importantly, these agents differ substantially in pharmacological maturity. Benzosulfonamide and cyanopyrrolidine-containing inhibitors provide structural evidence of direct engagement of the USP30 catalytic site, whereas activity-based probes enable the measurement of catalytically active USP30 and cellular target engagement [101,102,103]. Non-covalent inhibitors provide potentially reversible scaffolds for further pharmacological optimization [104]. However, most of these compounds were developed in non-oncology settings, and systematic comparisons of their potency, selectivity across the deubiquitinase family, pharmacokinetics, tumor penetration, and in vivo target engagement in cancer models remain limited.

Selective USP30 inhibition has now progressed into clinical development in non-oncology indications. MTX325, a brain-penetrant selective USP30 inhibitor, has completed early Phase I evaluation in healthy volunteers and is currently being evaluated in patients with Parkinson’s disease as part of a multi-part Phase I study (ISRCTN20898392) [105]. MTX652 has completed a first-in-human Phase I study in healthy volunteers and has received U.S. Food and Drug Administration (FDA) clearance for Phase II clinical evaluation in acute kidney injury [105,106]. These clinical milestones provide first-in-human support for the pharmacological tractability of USP30. Nevertheless, these programs are being developed outside oncology, and their clinical progress should not be interpreted as evidence of antitumor efficacy. Cancer-specific dosing, tumor target engagement, pharmacodynamic biomarkers, efficacy, and the relevant therapeutic window remain to be established (Table 2).

Several cancer-directed agents have provided proof-of-concept evidence, including MF-094 nanodelivery in oral squamous cell carcinoma, Aumdubin in lung cancer, the selenium-containing compound 8 m in prostate cancer, and sodium 2,2-dichloroacetate in colorectal cancer [18,22,23,56]. These studies support the pharmacological tractability of USP30-associated pathways in cancer, but they should not be regarded as equivalent demonstrations of selective USP30 inhibition. For some agents, broader target profiles and the extent of direct USP30 engagement remain to be clarified. Future studies should therefore combine antitumor efficacy with orthogonal evidence of target engagement, genetic rescue experiments, and substrate-specific pharmacodynamic readouts.

The therapeutic value of USP30 inhibition is also likely to depend on tumor-specific substrate dependency. Tumors with high USP30 activity or reliance on USP30-regulated proteins such as ACLY/FASN, HK2, FTO, DRP1, Snail, TOMM40, catalase, or GPX4 may be particularly susceptible to USP30 inhibition. In contrast, caution may be required in tumors in which nuclear USP30 restrains TCF/LEF1 signaling or in USP30-low glioblastoma-like settings. Biomarker development should integrate USP30 abundance and catalytic activity, substrate expression and ubiquitination status, mitochondrial phenotype, mitophagy competence, and immune-cell composition.

## 4. Mechanistic Gaps and Translational Priorities

The expanding substrate spectrum of USP30 raises several unresolved mechanistic and translational questions. First, the determinants of USP30 substrate specificity are poorly understood. USP30 was initially characterized through mitochondrial localization and its ability to regulate ubiquitin chains on mitochondrial substrates [2,3,5]. Structural studies further showed that USP30 preferentially cleaves Lys6-linked ubiquitin chains [30,31]. However, several cancer-associated substrates, including Snail, LEF1, FTO, and HK2, are not classical mitochondrial outer membrane substrates [17,18,24,26]. Whether USP30 directly deubiquitinates these proteins in distinct subcellular contexts or regulates them indirectly through mitochondria-dependent signaling remains unclear. For cancer-associated substrates that have been mechanistically characterized, current evidence primarily supports catalytic deubiquitination as the proximal mechanism of USP30 action. The downstream consequences can include changes in protein stability, localization, molecular interactions, enzymatic activity, and transcriptional regulation. Whether USP30 additionally exerts catalysis-independent scaffolding or regulatory functions in cancer remains insufficiently defined. Addressing this question will require catalytically inactive rescue experiments, separation-of-function mutants, proximity-labeling approaches, and direct measurement of substrate ubiquitination in the relevant cellular compartment.

Second, it remains unclear whether USP30 functions as a general oncogenic dependency or a context-specific regulator. Most mechanistic studies currently support tumor-promoting functions in hepatocellular carcinoma, colorectal cancer, oral squamous cell carcinoma, ovarian cancer, bladder cancer, prostate cancer, leukemia, and several breast cancer models, where USP30 sustains oncogenic or stress-adaptive proteins and pathways [16,17,18,19,20,21,22,23,24,25,57,58]. By contrast, tumor-restraining effects have been reported for nuclear USP30–TCF/LEF1 signaling in breast cancer and for USP30 overexpression in glioblastoma [26,27]. The strength of evidence is not equivalent in these cancers: the nuclear TCF/LEF1 mechanism has been defined at the substrate and transcriptional-complex levels, whereas the molecular basis of the glioblastoma phenotype remains unresolved. These observations suggest that the direction of USP30 activity is determined by the combination of tumor lineage, subcellular localization, substrate repertoire, cellular state, and environmental or therapeutic stress rather than by USP30 abundance alone. This distinction is particularly evident in breast cancer, where Snail- and TOMM40-associated activities are tumor-promoting, whereas nutrient stress-induced nuclear USP30 restrains TCF/LEF1-dependent transcription. Therapeutically, this context dependence argues against indiscriminate USP30 inhibition. Biomarker strategies may therefore need to assess not only USP30 expression or catalytic activity but also its subcellular distribution and the presence of functionally relevant substrates before defining a tumor as USP30-dependent.

Third, the relationship between PINK1/Parkin-dependent mitochondrial quality control and the broader cancer-associated functions of USP30 remains unresolved. USP30 inhibition enhances mitophagy in multiple experimental settings [11,12,13,15]. At the same time, cancer studies increasingly implicate USP30 in substrate-specific regulation of metabolism, EMT, stemness, and therapy resistance [16,17,18,20,24,26]. Whether these cancer-associated functions reflect altered mitophagy, mitochondrial stress signaling, or direct regulation of cancer-associated substrates remains an important mechanistic question.

Finally, the therapeutic window of USP30 inhibition in cancer remains undefined. Three translational issues require particular attention. First, available compounds should be benchmarked using standardized assays of biochemical potency, selectivity across the deubiquitinase family, cellular target engagement, and in vivo pharmacokinetics [101,102,103,104]. Second, inhibitors developed to enhance mitophagy in neurological or mitochondrial-disease models should be distinguished from agents with direct evidence of antitumor activity, as tissue exposure, dose requirements, and toxicity profiles may differ substantially [11,12,13,14,18,22,23,56]. Third, biomarker-guided patient-selection strategies are needed to identify tumors that are genuinely dependent on USP30-regulated substrates or mitochondrial stress adaptation.

Future studies should incorporate substrate-specific pharmacodynamic biomarkers and immunocompetent tumor models. Systemic USP30 inhibition may affect mitochondrial and peroxisomal quality-control pathways in non-malignant tissues, while also altering the fitness of exhausted T cells and other immune populations [12,13,32,33,59]. Defining the balance between tumor-cell vulnerability, immune-cell reinvigoration, and normal-tissue organelle homeostasis will therefore be essential for establishing an acceptable therapeutic window.

These unanswered questions highlight an important point: USP30 should not yet be viewed as a universally validated cancer target. Rather, it should be considered an emerging, mechanistically plausible, and pharmacologically tractable deubiquitinase whose therapeutic value will depend on careful definition of cancer-type specificity, substrate dependency, cellular context, and drug properties.

## 5. Conclusions

USP30 was originally defined as a mitochondrial outer membrane-localized deubiquitinase that regulates mitochondrial morphology and antagonizes mitophagy [2,3]. Although its established role in PINK1/Parkin-dependent mitophagy remains central to USP30 biology, this function alone does not fully explain the expanding functions of USP30 in cancer. Accumulating evidence indicates that USP30 can regulate diverse cancer-associated processes, including metabolic adaptation, mitochondrial dynamics, apoptosis, ferroptosis, EMT, cancer stemness, therapeutic resistance, and tumor immunity [16,17,18,19,20,24,26].

Rather than acting through a single linear pathway, USP30 appears to function as a context-dependent regulator whose effects are shaped by tumor type, subcellular localization, substrate availability, mitochondrial state, and immune microenvironment. In many tumor models, USP30 supports oncogenic adaptation through substrate-specific deubiquitination. The resulting effects can extend beyond protein stabilization to include changes in subcellular localization, molecular interactions, enzymatic activity, transcriptional-complex assembly, and stress signaling. However, in some tumors or cellular states, USP30 may also exert tumor-restraining effects, emphasizing that its biological functions cannot be generalized across cancers.

From a therapeutic perspective, USP30 represents an attractive but incompletely validated oncology target. Its enzymatic activity and defined catalytic architecture make it pharmacologically tractable, and several inhibitors, chemical probes, and cancer-directed agents have been reported. Nevertheless, future development will require clearer evidence of selective target engagement, cancer-specific pharmacodynamics, biomarker-defined sensitivity, and an acceptable therapeutic window. It will also be important to determine whether the antitumor effects of USP30 inhibition arise mainly from enhanced mitophagy, destabilization of cancer-promoting substrates, altered mitochondrial stress signaling, immune modulation, or a combination of these mechanisms.

Overall, the understanding of USP30 has evolved from that of a predominantly mitophagy-associated mitochondrial deubiquitinase to that of a spatially and functionally diverse regulator of cancer-associated ubiquitin signaling. USP30 does not simply act as a suppressor of mitophagy; it can also influence cancer adaptation through the substrate-selective regulation of diverse cancer-associated proteins. Defining when and how tumors depend on USP30-regulated substrates or mitochondrial stress adaptation will be critical for translating USP30 biology into rational cancer therapies.

## Figures and Tables

**Table 1:** Cancer-associated substrates and signaling pathways regulated by USP30.

Cancer Context	USP30 Substrates or Signaling Axis	Mechanistic Regulation	Functional Consequence	Overall Effect	Ref.
Multiple cancer models	Parkin-dependent mitochondrial substrates	USP30 removes Parkin-dependent ubiquitin signals from outer mitochondrial membrane proteins and limits mitochondrial apoptotic signaling.	Increased apoptotic threshold and reduced sensitivity to BH3 mimetics.	Tumor-promoting	[6]
Hepatocellular carcinoma	GNPAT–USP30–DRP1 axis	GNPAT recruits USP30 to DRP1, promoting DRP1 deubiquitination and stabilization.	Mitochondrial fission, proliferation, and hepatocarcinogenesis.	Tumor-promoting	[19]
	IKKβ–USP30–ACLY/FASN axis	IKKβ phosphorylates USP30 at Ser210 and Ser364, increasing USP30 stability; USP30 deubiquitinates and stabilizes ACLY and FASN.	Enhanced lipogenesis, inflammatory signaling, and tumor growth.	Tumor-promoting	[16]
Breast cancer	USP30–Snail axis	USP30 removes K48-linked polyubiquitin chains from Snail and increases its stability.	EMT, invasion, metastasis, tumor growth, and paclitaxel resistance.	Tumor-promoting	[24]
	USP30–TOMM40 axis	USP30 deubiquitinates and stabilizes TOMM40.	Mitochondrial adaptation and tamoxifen resistance.	Tumor-promoting	[20]
	mTORC1–USP30–TCF/LEF1 axis	Under nutrient stress, nuclear USP30 deubiquitinates TCF/LEF1 at K379 and K382 and impairs CBP/P300 recruitment.	Suppression of Wnt-driven stemness, metastasis, and adriamycin resistance.	Tumor-restraining	[26]
Lung cancer	USP30–targeted pharmacological inhibition	Aumdubin inhibits USP30 and induces BAX-dependent mitochondrial apoptosis.	Suppressed tumor-cell survival in vitro and in vivo.	Tumor-promoting	[56]
Oral squamous cell carcinoma	USP30–c-Myc axis	USP30-dependent deubiquitination stabilizes c-Myc.	Increased cell viability and glutamine consumption, and resistance to apoptosis.	Tumor-promoting	[23]
Leukemia-related model	USP30–Parkin–AKT/mTOR	USP30 antagonizes Parkin activity and sustains AKT/mTOR signaling.	Reduced apoptosis and enhanced survival signaling.	Tumor-promoting	[57]
Acute myeloid leukemia	USP30–FOXM1	USP30 interacts with FOXM1 and deubiquitinates and stabilizes FOXM1 by limiting its proteasomal degradation.	Enhanced proliferation and cell-cycle progression, reduced apoptosis, and increased cytarabine resistance; USP30 depletion suppresses growth of cytarabine-resistant AML cells in vivo.	Tumor-promoting	[58]
Bladder cancer	Parkin–USP30–Catalase axis	USP30 directly deubiquitinates and stabilizes catalase, counteracting Parkin-dependent ubiquitination.	Reduced intracellular ROS and increased proliferation and migration.	Tumor-promoting	[21]
Ovarian cancer	FOXF1–HMGA2–USP30– S100A6 axis	FOXF1 increases HMGA2, which recruits USP30 to S100A6; USP30 deubiquitinates and stabilizes S100A6.	Cytoskeletal remodeling, migration, and metastatic dissemination.	Tumor-promoting	[25]
Prostate cancer	USP30–GPX4 axis	USP30 deubiquitinates and stabilizes GPX4; USP30 inhibition increases GPX4 ubiquitination and degradation.	Reduced lipid peroxidation and ferroptosis resistance.	Tumor-promoting	[22]
Multiple cancer models	USP30–HK2 axis	USP30 regulates HK2 ubiquitination at K144 and increases HK2 stability, mitochondrial localization, and VDAC1 interaction.	Enhanced glycolysis and tumor progression.	Tumor-promoting	[17]
Colorectal cancer	USP30–FTO–PHGDH/PSAT1 axis	USP30 deubiquitinates and stabilizes FTO, thereby activating the PHGDH/PSAT1 serine-biosynthesis program.	Serine biosynthesis and adaptation to nutrient stress.	Tumor-promoting	[18]
Tumor-infiltrating CD8+ T cells	TCR/NFAT1–USP30–mitophagy	Chronic TCR stimulation induces USP30 through NFAT1 and suppresses mitophagy in exhausted CD8+ T cells.	Reduced mitochondrial fitness and antitumor effector function; USP30 inhibition restores T-cell activity.	Immune-suppressive	[59]
Glioblastoma	Undefined substrate/mechanism	USP30 is downregulated in tumors; USP30 overexpression inhibits growth-related phenotypes in U87 cells.	Reduced proliferation, DNA synthesis, and colony formation.	Tumor-restraining	[27]

BH3, BCL-2 homology 3; GNPAT, glyceronephosphate O-acyltransferase; DRP1, dynamin-related protein 1; IKKβ, inhibitor of nuclear factor kappa-B kinase subunit beta; ACLY, ATP citrate lyase; FASN, fatty acid synthase; EMT, epithelial–mesenchymal transition; TOMM40, translocase of outer mitochondrial membrane 40; mTORC1, mechanistic target of rapamycin complex 1; TCF/LEF1, T-cell factor/lymphoid enhancer-binding factor 1; CBP, CREB-binding protein; BAX, BCL-2-associated X protein; AKT, protein kinase B; mTOR, mechanistic target of rapamycin; FOXM1, forkhead box M1; AML, acute myeloid leukemia; ROS, reactive oxygen species; FOXF1, forkhead box F1; HMGA2, high-mobility group AT-hook 2; S100A6, S100 calcium-binding protein A6; GPX4, glutathione peroxidase 4; HK2, hexokinase 2; VDAC1, voltage-dependent anion channel 1; FTO, fat mass and obesity-associated protein; PHGDH, phosphoglycerate dehydrogenase; PSAT1, phosphoserine aminotransferase 1; TCR, T-cell receptor; NFAT1, nuclear factor of activated T cells 1.

**Table 2:** Representative USP30 inhibitors and their preclinical and clinical development.

Compound/Agent	USP30-Targeting Evidence/Modality	Development Stage	Disease/Experimental Context	Key Findings and Current Limitations	Ref.
MTX325	Selective, brain-penetrant small-molecule USP30 inhibitor	Phase I	Parkinson’s disease; healthy volunteers and patients	Phase I clinical development supports the pharmacological tractability of USP30 in humans; no oncology efficacy or tumor target-engagement data.	[105]
MTX652 (MTX115652)	Selective small-molecule USP30 inhibitor	Phase I completed; Phase II cleared	Acute kidney injury	Completed a first-in-human Phase I study in healthy volunteers and received FDA clearance for Phase II clinical evaluation in acute kidney injury. No oncology clinical data are currently available.	[105,106]
FT3967385 (FT385)	N-cyanopyrrolidine covalent tool inhibitor with high USP30 selectivity at low concentrations	Preclinical	Mitophagy and neuronal models	Increases TOM20 ubiquitination and enhances mitophagy; off-target effects emerge at higher exposure.	[35]
Compound 39	Benzosulfonamide inhibitor with direct USP30 catalytic-site engagement	Preclinical	Mitophagy and pexophagy models	Enhances mitophagy and basal pexophagy with strong USP30 engagement; no oncology clinical data.	[12,101]
MF-094	Small-molecule USP30 inhibitor; nanoparticle delivery evaluated in cancer	Preclinical	Oral squamous cell carcinoma	Nanodelivery suppresses viability, glutamine consumption, and tumor growth in vitro and in vivo; no clinical validation.	[11,23]
Aumdubin	Gold(I)-containing auranofin derivative with affinity for USP30 and other DUBs	Preclinical	Lung cancer	Induces BAX-dependent mitochondrial apoptosis and suppresses xenograft growth; multi-DUB activity limits USP30-specific attribution.	[56]
Compound 8 m	Selenium-containing small molecule reported to inhibit USP30	Preclinical	Advanced prostate cancer	Promotes GPX4 ubiquitination/degradation and ferroptosis; broader USP30 selectivity requires validation.	[22]
DCA	Sodium 2,2-dichloroacetate; repurposed small molecule identified as a USP30 inhibitor	Preclinical	Colorectal cancer	Suppresses the USP30–FTO–PHGDH/PSAT1 serine-biosynthesis program and tumor growth; selectivity and target engagement require validation.	[18]

DUB, deubiquitinase; FDA, U.S. Food and Drug Administration; TOM20, translocase of outer mitochondrial membrane 20.

## Data Availability

No new data were created or analyzed in this study. Data sharing is not applicable to this article.

## Abbreviations

The following abbreviations are used in this manuscript:

USP30	ubiquitin-specific protease 30
PINK1	PTEN-induced kinase 1
EMT	epithelial–mesenchymal transition
ACLY	ATP citrate lyase
FASN	fatty acid synthase
HK2	hexokinase 2
FTO	fat mass and obesity-associated protein
TCF/LEF1	T-cell factor/lymphoid enhancer-binding factor 1
GPX4	glutathione peroxidase 4
DRP1	dynamin-related protein 1
TOMM40	translocase of outer mitochondrial membrane 40
S100A6	S100 calcium-binding protein A6
IKKβ	inhibitor of nuclear factor kappa-B kinase subunit beta
mTORC1	mechanistic target of rapamycin complex 1
MFN1/2	mitofusin 1/2
GNPAT	glyceronephosphate O-acyltransferase
PEX2	peroxisomal biogenesis factor 2
HCC	hepatocellular carcinoma
VDAC1	voltage-dependent anion channel 1
PHGDH	phosphoglycerate dehydrogenase
PSAT1	phosphoserine aminotransferase 1
DCA	sodium 2,2-dichloroacetate
FOXF1	forkhead box F1
HMGA2	high-mobility group AT-hook 2
ROS	reactive oxygen species
TCR	T-cell receptor
